# Transforming dysfunctional CD8^+^ T cells into natural controller–like CD8^+^ T cells: can TCF-1 be the magic wand?

**DOI:** 10.1172/JCI160474

**Published:** 2022-06-01

**Authors:** Hiroshi Takata, Lydie Trautmann

**Affiliations:** Vaccine and Gene Therapy Institute, Oregon Health and Science University, Beaverton, Oregon, USA.

## Abstract

HIV infection results in defective CD8^+^ T cell functions that are incompletely resolved by antiretroviral therapy (ART) except in natural controllers, who have functional CD8^+^ T cells associated with viral control. In this issue of the *JCI*, Perdomo-Celis et al. demonstrated that targeting the Wnt/transcription factor T cell factor 1 (Wnt/TCF-1) pathway in dysfunctional CD8^+^ T cells led to gains in stemness phenotype, metabolic quiescence, survival potential, response to homeostatic γ-chain cytokines, and antiviral capacities, similar to profiles of functional CD8^+^ T cells in natural controllers. Although reprogramming might not sufficiently reverse the imprinted dysfunction of CD8^+^ T cells in HIV infection, these findings outline the Wnt/TCF-1 pathway as a potential target to reprogram dysfunctional CD8^+^ T cells in efforts to achieve HIV remission.

## Targeting TCF-1 to reprogram dysfunctional CD8^+^ T cells

Harnessing HIV-specific CD8^+^ T cell responses is explored as a strategy to induce HIV remission, since these cells play a critical role in limiting viral replication and eliminating HIV-infected cells in acute infection (1, 2). Functional HIV-specific CD8^+^ T cell responses have also been associated with viral control in natural controllers (3). However, in most people, dysfunction of HIV-specific CD8^+^ T cells begins after peak viremia in acute infection (4) and persists in the absence of treatment (5). Initiation of antiretroviral therapy (ART) only partially restores CD8^+^ T cell functions, therefore, it is critical for HIV remission strategies to reinvigorate HIV-specific CD8^+^ T cells in people living with HIV.

During an acute infection in the mouse model, primed CD8^+^ T cells differentiate into a large subset of short-lived effector cells and a small subset of memory precursor cells that give rise to long-lived memory cells (6). Transcription factor T cell factor 1 (TCF-1) is downstream of the Wnt/β-catenin pathway and involved in thymocyte maturation and T cell development (7). TCF-1 also participates in the transcriptional program for memory CD8^+^ T cell differentiation, longevity, and secondary expansion (8). Naive CD8^+^ T cells express high levels of TCF-1, which contrasts with effector CD8^+^ T cells, in which TCF-1 is downregulated. High TCF-1 levels in a small fraction of antigen-experienced CD8^+^ T cells allows these memory precursor cells to acquire a stem cell–like phenotype and to persist long term. In 2009, Gattinoni et al. demonstrated that promoting the Wnt/β-catenin pathway drives the generation of stem cell–like memory cells (9). Later, Youngblood et al. showed that effector CD8^+^ T cells can change their epigenetic landscape and dedifferentiate into long-lived memory cells (10), suggesting that researchers can generate long-lived memory cells with stem cell properties. Studies in the lymphocytic choriomeningitis virus (LCMV) model have shown that TCF-1 not only plays a crucial role during CD8^+^ T cell memory formation and maintenance, but also in the regulation of CD8^+^ T cell exhaustion in chronic infection. Indeed, TCF-1^+^ CD8^+^ T cells in chronic infection have the ability to self-renew and retain their proliferative potential, even though they express checkpoint proteins such as programmed cell death 1 (PD-1). These stem cell–like exhausted cells in chronic infection give rise to and constantly replenish the pool of low TCF-1–expressing, short-lived, exhausted CD8^+^ T cells, hence the term “precursor exhausted” cells (11–13). Notably, ectopic expression of TCF-1 in exhausted effector CD8^+^ T cells reprograms them into precursor exhausted cells (14). These data suggest that targeting the TCF-1 pathway can reprogram exhausted effector CD8^+^ T cells into CD8^+^ T cells with stem cell–like properties. In this issue of the *JCI*, Perdomo-Celis et al. used a pan–glycogen synthase kinase 3 (GSK-3) inhibitor, targeting the TCF-1/Wnt/β-catenin pathway to induce TCF-1 and its transcriptional activity, which reprogrammed dysfunctional CD8^+^ T cells in HIV infection (15).

## Reprogramming differentiation toward stem cell–like memory CD8^+^ T cells

**Figure 1:**
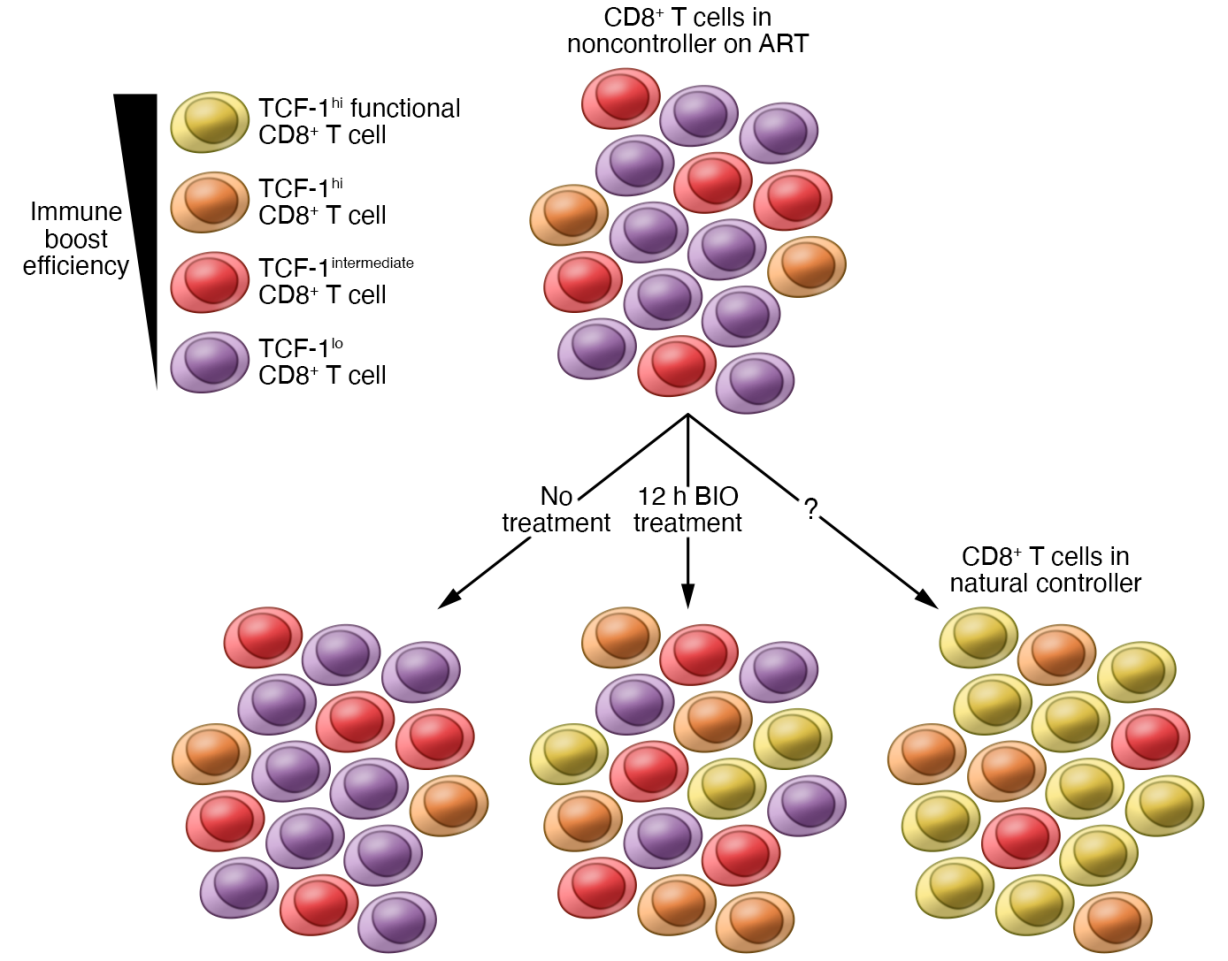
Features of HIV-specific CD8^+^ T cells in natural controllers and noncontrollers on ART with and without reprogramming through GSK-3 inhibition. CD8^+^ T cells from natural controllers consist of a high frequency of functional, long-lived memory HIV-specific cells expressing TCF-1 at high levels, whereas those from noncontrollers on ART consist of a low frequency of HIV-specific cells that express low or intermediate levels of TCF-1 and exhibit residual dysfunction. Treatment with the GSK-3 inhibitor BIO for 12 hours increased the expression of TCF-1 in these cells. Short-term GSK-3 inhibition of HIV-specific CD8^+^ T cells in noncontrollers improves their metabolic fitness, survival capacity, homeostatic proliferation, and antiviral capacity, although these features might still be less prominent than those in natural controllers. Reprogramming HIV-specific CD8^+^ T cells by GSK-3 inhibition to increase stemness may allow for more efficient immune boosting, resulting in a higher number of HIV-specific CD8^+^ T cells with enhanced survival and antiviral capacities to help control the virus after treatment interruption.

Although TCF-1 has been extensively studied in the mouse model, only a few human studies have identified TCF-1 as a key transcription factor expressed in self-renewing memory CD8^+^ T cells during viral infections (16, 17). In people with hepatitis C virus (HCV) infection, TCF-1–expressing, HCV-specific CD8^+^ T cells persist after viral clearance (18). HIV-specific CD8^+^ T cells in natural controllers that have a preserved proliferative capacity exhibit elevated TCF-1 expression compared with noncontrollers (17, 19). Perdomo-Celis et al. demonstrated that the GSK-3 inhibitor 6-bromoindirubin-3′-oxime (BIO) was able to promote TCF-1 upregulation within a portion of total and HIV-specific CD8^+^ T cells after 12 hours of treatment, resulting in their dedifferentiation into stem cell memory T cell (TSCM) and central memory T cell (TCM) phenotypes. Upon T cell receptor (TCR) stimulation, BIO-treated CD8^+^ T cells had increased expression of BCL-6, CD127, and genes associated with cell survival (including *BCL2*), reflective of their long-lived memory potential. The cells also had decreased expression of PD-1, T-bet, B lymphocyte–induced maturation protein 1 (BLIMP-1), and genes associated with effector (*IFNG*, *BATF*, *IFNGR1*, and *GZMK*) and exhausted (*CD244* and *HAVCR2*, which encodes T cell Ig and mucin domain–containing protein 3 [TIM-3]) T cells. Additionally, Perdomo-Celis et al. demonstrated that BIO treatment also improved the homeostatic proliferation of total and HIV-specific CD8^+^ T cells in response to IL-15 and IL-7, further showing that TCF-1 induction promoted the reprogramming of dysfunctional CD8^+^ T cells into cells with a higher persistence capacity (Figure 1). The authors did not show that BIO treatment improved TCR-mediated proliferation, although we would expect cells with a higher survival potential to respond better to TCR triggering (15).

## Reprogramming cell metabolism toward quiescent memory CD8^+^ T cells

Increasing evidence supports the role of metabolic pathways in shaping immune responses and outcomes during HIV infection (20). Effector CD8^+^ T cells in acute HIV infection upregulate the mechanistic target of rapamycin complex 1 (mTORC1) and aerobic glycolysis to sustain the energy and biomass increase needed for their proliferation and function. HIV-specific CD8^+^ T cells in uncontrolled viremia exclusively depend on glycolysis and are maintained by continuous proliferation (21). In contrast, HIV-specific CD8^+^ T cells found in HIV natural controllers have more metabolic plasticity and rely on both glycolysis and mitochondrial respiration to survive and exert their function. The spontaneous loss of viral control in natural controllers is preceded by a decrease in the functionality of HIV-specific CD8^+^ T cells associated with an increase in plasma markers of aerobic glycolysis, dysregulated mitochondrial activity, and oxidative stress (22). A previous report showed that GSK-3 regulates mTORC1 activity by phosphorylating the mTOR-associated scaffold protein Raptor (23). Perdomo-Celis et al. demonstrate that GSK-3 inhibition reprogrammed total CD8^+^ T cells into metabolically more quiescent CD8^+^ T cells by reducing glucose and lipid uptake, mitochondrial mass, and the level of ROS. They confirmed that the metabolic plasticity was also increased in HIV-specific CD8^+^ T cells, as these cells decreased mTORC1-driven anabolic metabolism, similar to long-lived memory CD8^+^ T cells or HIV-specific CD8^+^ T cells in natural controllers (Figure 1 and ref. 15).

## Effector functions after CD8^+^ T cell reprogramming

The purpose of reprogramming dysfunctional CD8^+^ T cells in HIV remission strategies is to enable them to eliminate HIV-infected cells and/or reduce viral transcription. TCF-1 has been reported to negatively correlate and repress the expression of genes involved in cytolytic programs and inhibit the differentiation of effector cells (13, 19). However, Perdomo-Celis et al. demonstrated that BIO treatment of CD8^+^ T cells increased TNF-α secretion and resulted in polyfunctional T cells with reduced expression of checkpoint proteins (Figure 1). BIO treatment also enhanced viral suppression in an in vitro HIV replication assay without upregulation of cytolytic effector molecules (15). The mechanism leading to the exclusive increase of TNF-α production in the reprogrammed cells, mainly responsible for the increase in polyfunctional cells, still needs to be elucidated. The superior antiviral activity might, therefore, result from increased TNF-α secretion or inhibition of HIV transcription rather than enhanced cytolytic activity. Another explanation for the discordant repression of effector genes and enhanced viral suppression might lie in the timing of BIO treatment: induction of TCF-1 with a short BIO exposure prior to TCR triggering might lead to enhanced differentiation of these reprogrammed cells into potent effector cells with downregulation of TCF-1, whereas continuous BIO exposure might maintain elevated TCF-1 levels in CD8^+^ T cells, thereby preventing effector differentiation. Therefore, reprogramming dysfunctional CD8^+^ T cells without interfering with their effector abilities may require sequential therapeutic strategies.

## Targeting TCF-1 has therapeutic implications

One main difference between HIV-specific CD8^+^ T cells in natural controllers and noncontrollers on ART, besides their survival and cytolytic capacities, is their quantity; natural controllers have higher numbers of HIV-specific CD8^+^ T cells than do noncontrollers. However, therapeutic vaccinations and other immunotherapies aimed at boosting HIV-specific CD8^+^ T cells in noncontrollers on ART have so far induced a nominal delay of viral rebound after analytical treatment interruption (24). One possible cause for this lack of efficacy is the residual CD8^+^ T cell dysfunction that persists while the individual is on ART, which renders these cells unable to respond properly to immunotherapies. Recent studies in a murine cancer model and chronic LCMV infection demonstrated that TCF1^+^ precursor exhausted CD8^+^ T cells comprise a specific fraction of CD8^+^ T cells that proliferate after PD-1 blockade therapy to form a large pool of short-lived cytotoxic effectors (25). The response to PD-1 blockade therapy is highly variable and the reasons for this variability remain unclear, but melanoma patients with melanoma who have a higher proportion of TCF-1^+^ CD8^+^ T cells showed a better response after PD-1 blockade and survived longer (26). Given these data, TCF-1 becomes an attractive therapeutic target that may be key for immunotherapies in people with residual CD8^+^ T cell dysfunction. Even if a small portion of HIV-specific CD8^+^ T cells are reprogrammed into long-lived memory cells that proliferate and differentiate into a large pool of effector cells, this small subset of cells might provide effective immune responses (Figure 1). Successful HIV remission strategies would likely require a combination strategy aimed first at reprogramming HIV-specific CD8^+^ T cells to express higher TCF-1 levels and then boosting them to expand prior to analytical treatment interruption. Combination strategies could include a small molecule promoting the transcriptional activity of TCF-1 and any immune-boosting strategies that are already undergoing testing, including the use of mRNA vaccines, immune checkpoint inhibitors, IL-15 superagonists, or chimeric antigen receptor (CAR) T cells. Combining TCF-1 induction with any of these immune interventions has the potential to enhance their efficacy.

## Remaining questions to be answered

Although the data published by Perdomo-Celis et al. (15) provide a potential target to reprogram dysfunctional CD8^+^ T cells in efforts to achieve HIV remission, several questions remain unanswered. The authors showed that short-term GSK-3 inhibition reprogrammed a fraction of HIV-specific CD8^+^ T cells toward cells with a stem cell–like phenotype, metabolic plasticity, survival potential, response to homeostatic γ-chain cytokines, and antiviral capacity. However, no direct comparison was made with functional HIV-specific CD8^+^ T cells from natural controllers, and it is still unknown whether the reprogrammed HIV-specific CD8^+^ T cells compare functionally with those in natural controllers. Additionally, the level of TCF-1 expression and the fraction of cells that require the regaining of TCF-1 expression to ultimately eliminate HIV-infected cells and control viral transcription still need to be defined. Studies have shown that TCF-1 is a transcription factor that can directly modify histone acetylation, linking transcriptional and epigenetic regulation. Exhausted CD8^+^ T cells in HIV infection acquire a distinct epigenetic state, but whether BIO treatment and TCF-1 induction change that chromatin landscape remains unknown. It will be important to perform epigenetic analyses to define reversions in the chromatin landscape associated with exhaustion. Indeed, in HCV infection, HCV-specific CD8^+^ T cells possess a largely irreversible epigenetic program of exhaustion, even after the infection resolves, suggesting that CD8^+^ T cells maintain the molecular signature as a chronic scar (27, 28). It will also be important to determine whether the dedifferentiation of CD8^+^ T cells is temporary or permanent. Further experiments will also need to fine-tune the timing and duration of treatment with GSK-3 inhibitors required to enhance TCF-1 expression, while preventing the inhibition of HIV-specific CD8^+^ T cell expansion and effector differentiation in vivo. A deeper understanding of the mechanism by which GSK-3 inhibition via BIO treatment leads to the upregulation of TCF-1 would allow for the selection of more specific small molecules with potentially lower off-target effects than a pan–GSK-3 inhibitor. Finally, further experiments should explore the effect of TCF-1 induction on transcriptional regulation of the HIV provirus, as TCF-1 could induce latent reservoir reactivation (29, 30). While it is premature to say that TCF-1 is the magic wand that can transform dysfunctional CD8^+^ T cells into natural controller–like functional CD8^+^ T cells, targeting the Wnt/TCF-1 pathway to reprogram CD8^+^ T cells to increase stemness might provide a necessary component for HIV remission–inducing immunotherapy.

## Version 1 06/01/2022

Electronic publication
